# Comparative Study of Different Ion-Exchange Membrane Types in Diffusion Dialysis for the Separation of Sulfuric Acid and Nickel Sulfate

**DOI:** 10.3390/membranes13040396

**Published:** 2023-03-30

**Authors:** Sergey Loza, Natalia Loza, Nikita Kovalchuk, Nazar Romanyuk, Julia Loza

**Affiliations:** Physical Chemistry Department, Faculty of Chemistry and High Technologies, Kuban State University, 350040 Krasnodar, Russia

**Keywords:** diffusion dialysis, ion-exchange membrane, diffusion permeability, sulfuric acid, nickel sulfate, water flux, osmosis, water permeability

## Abstract

The possibility of using various types of ion-exchange membranes in diffusion dialysis for the separation of sulfuric acid and nickel sulfate has been evaluated. The process of the dialysis separation of a real waste solution from an electroplating facility containing 252.3 g/L of sulfuric acid, 20.9 g/L of nickel ions and small amounts of zinc, iron, copper ions, etc. has been studied. Heterogeneous cation-exchange membrane containing sulfonic groups and heterogeneous anion-exchange membranes with different thicknesses (from 145 μm to 550 μm) and types of fixed groups (four samples with quaternary ammonium base and one sample with secondary and tertiary amines) have been used. The diffusion fluxes of sulfuric acid, nickel sulfate, and the total and osmotic fluxes of the solvent have been determined. The use of a cation-exchange membrane does not allow the separation of the components, since the fluxes of both components are low and comparable in magnitude. The use of anion-exchange membranes makes it possible to efficiently separate sulfuric acid and nickel sulfate. Anion-exchange membranes with quaternary ammonium groups are more effective in the diffusion dialysis process, while the thin membrane turns out to be the most effective.

## 1. Introduction

The concept of sustainable development is formulated in the Report of the World Commission on Environment and Development: Our Common Future “Sustainable development is the development that meets the needs of the present without compromising the ability of future generations to meet their own needs” [1]. However, the growth of industrial and agricultural activities leads to an increase in various kinds of waste, including wastewater with toxic compounds. The development of Minimal and Zero Liquid Discharge (MLD and ZLD, respectively) systems facilitates the reduction of negative environmental impacts and the recovery of resources. Various technologies for MLD and ZLD systems are discussed in a number of reviews [2,3,4,5,6]. MLD and ZLD can include electrochemical coupling technologies, adsorption, flotation, extraction, ion-exchange methods, etc. Membrane technologies are also promising for these systems [7,8]. The recovery of metal compounds and acids from the wastewater of metallurgical plants is the one of the most important problems solved by membrane technologies.

In general, dialysis is a process of the membrane separation of solution components in which transport is driven primarily by concentration differences [9]. If an ion-exchange membrane is used in the dialysis, such a process is called diffusion dialysis (DD) [10]. DD is a perspective method used for the separation of organic and inorganic components [11,12] and the extraction of metal ions from mixtures [13]. Examples of DD implementation in technology include nickel recuperation in the process of lithium-ion accumulator recycling [14], nickel extraction from a used catalyst [15], and the separation of nonferrous metal salts from acids [10], including separation during galvanic wastewater processing [16,17,18,19,20,21]. Using an anion-exchange membrane (AEM) for separating nickel sulfate and sulfuric acid enables the extraction of about 66–72% of sulfuric acid, depending on the conditions of the dialysis procedure [10,22].

Since the driving force in DD is the concentration gradient of the solution components, there is no need for an external electric field for its application, which significantly reduces energy consumption compared to electrodialysis (ED) [10]. Simple and low-cost equipment is another advantage of DD over ED. Neither a DC power supply nor polarizing electrodes, which are often made from expensive materials such as platinized titanium, are required for DD. The processing of wastewaters originating from galvanic plants is relevant both in terms of decreasing environmental pollution and in terms of saving non-renewable resources by extracting them and returning them to production. However, there is the problem of achieving sufficient performance for the wide industrial application of DD. The driving force of the process is limited by the concentration of the feed solution and cannot be increased by an external influence, resulting in a relatively low capability [10]. The efforts of the developers of the DD modules aim to increase the area of the membrane surface in contact with the feed solution. The use of spiral-wound modules instead of plate-and-frame ones allows this problem to be solved [18]. The use of profiled membranes instead of flat ones also improves the efficiency of DD [12].

The efficiency of diffusion dialysis depends on such membrane properties as thickness, moisture content, ion-exchange capacity, porosity, selectivity, and diffusion permeability [13,23]. In addition, work is underway to create new dialysis membranes with optimal structure and properties, including surface hydrophilicity and water content [24,25,26]. Although there are quite a few commercial membranes designed for use in DD, it is possible to use conventional electrodialysis membranes in this process [12,27]. Some dialysis membranes and modules are not recommended for processing solutions containing organic components [28]. At the same time, conventional heterogeneous electrodialysis membranes can be successfully used in some organic solvents [29,30]. However, such membranes are much thicker compared with dialysis membranes. In [27], the authors show the efficiency of using the electrodialysis Ralex AMHPES membrane, which turned out to be effective for the preconcentration of phosphate from industrial wastewater by DD. Despite the fact that it is not the most efficient, the authors note the promise in using this membrane, including for economic reasons. Thus, evaluating the impact of using different types of membranes in the DD process in the same experimental conditions is of interest.

In this regard, the study aims to evaluate the efficiency and practical applicability of DD for separating nickel sulfate and sulfuric acid using various types of ion-exchange membranes (IEMs). Only industrially produced IEMs are used for DD. At the same time, the efficiency of using both dialysis IEMs and conventional electrodialysis IEMs in DD will be given. In addition, all experiments are performed on a real waste solution of an electroplating facility. The main objective of the study is to obtain a nickel sulfate solution with a sulfuric acid concentration of less than 0.1–0.2 mol/L.

## 2. Materials and Methods

### 2.1. Ion-Exschange Membranes

All conventional electrodialysis IEMs were heterogeneous and consisted of an inert binder (polyethylene) and particles of ion-exchange resin [31,32,33] (samples 1–5, Table 1). Heterogeneous AEMs made on the basis of strongly basic anion-exchange aminated polystyrene resins cross-linked with divinylbenzene (samples 1, 2, 5, Table 1) and a heterogeneous cation-exchange membrane (CEM) based on sulfonated polystyrene cross-linked with divinylbenzene (sample No. 3) were used. In addition, a weak basic AEM (sample No. 4) and a thin dialysis anion-exchange membrane (sample No. 6) were studied.

The membranes had different thicknesses, reinforcing meshes, ionogenic group types, and ion-exchange capacities (Table 1). Samples No. 1–3 were provided by MEGA a. s. (Prague, Czech Republic), samples No. 4, 5 by LLC Innovation Enterprise Shekinoazot (Tula, Russia), and sample No. 6 by Tianwei Membrane Corporation Ltd. (Weifang High-tech Zone, Shandong, China). IEMs were conditioned according to a standard procedure described in detail in [36] and in Appendix A. Procedures for determining the ion-exchange capacity (*Q*, mmol/g_wet_) and water content (*W*, %) are described in Appendix A. Thickness (*l*, μm) and water content in Table 1 were given in the H^+^-form for CEMs and in the form of sulfate anions for AEMs.

The thickness of membranes changed in series Ralex AMHPES = Ralex AMHPP > Ralex CMHPES ≈ MA-40 > MA-41 >> TWDDA3 (Table 1). The Ralex AMHPES and Ralex AMHPP had similar properties and compositions except for the reinforcing mesh [31]. The Ralex AMHPES, Ralex AMHPP, and MA-41 membranes had similar compositions. The main difference between the MA-41 membrane and the Ralex AMHPES and Ralex AMHPP membranes was the particle size of the ion-exchange resin. The particle sizes of ion-exchange resin in the MA-40 and MA-41 membranes were about 10–40 μm [38,39]. The particle sizes of ion-exchange resin in the Ralex membranes were 0.4–19 μm [40,41]. The portion of conducting regions on the MA-40 and MA-41 membranes’ surfaces was 0.16–0.28 [32,42]. The distance between the particles of ion-exchange resin on the surface of the Ralex membranes was less than for the MA-40 and MA-41 membranes due to the difference in particle sizes. The appearance of micro- and mesopores in the structure of the heterogeneous membranes was found by the standard contact porosimetry method [43,44]. The effective radii of the mesopores were 10–1000 nm. The mesopores were located between particles of ion-exchange resin and inert binder. The effective micropore radii was lower than 10 nm and contained the ion–dipole associates of fixed ion–counter-ion and their hydration shells. In general, differences in the structure of heterogeneous membranes were not significant.

The *k_i_*_so_ (Table 1) parameter was the conductivity of the gel phase of an IEM. According to the microheterogeneous model [44,45], the IEMs consisted of two conductive phases. One of them was called the solution or intergel phase and was the same as the external solution bathing the membrane. Another conductive phase was called the gel phase and included the ionogenic groups with the fixed- and counterions, their water hydration, polymer matrix and inert binder. The conductivity of the gel phase was the constant and depended on the ion exchange capacity of the membrane and the nature of the counter-ion. This parameter was determined as an intersection point of the concentration dependence of the conductivity of the solution and the membrane. The independence of this value from the concentration of the electrolyte solution made it possible to compare the conductive properties of different membranes. The value of *k*_iso_ was determined for the sodium chloride solution because it was the most commonly used solution for membrane characterization. The *k*_iso_ parameter of the MA-41 membrane were lower than that of the others due to the MA-41 membrane having the lowest ion exchange capacity. As for the MA-40 membrane, the low value of *k*_iso_ was due to the large number of tertiary and secondary amino groups that did not participate in ion exchange in neutral or alkaline solutions. The Ralex membranes had similar *k*_iso_ values. The selectivity of the conventional electrodialysis IEMs, described by the counter-ion transport number, were similar (Table 1).

**Figure 1:**
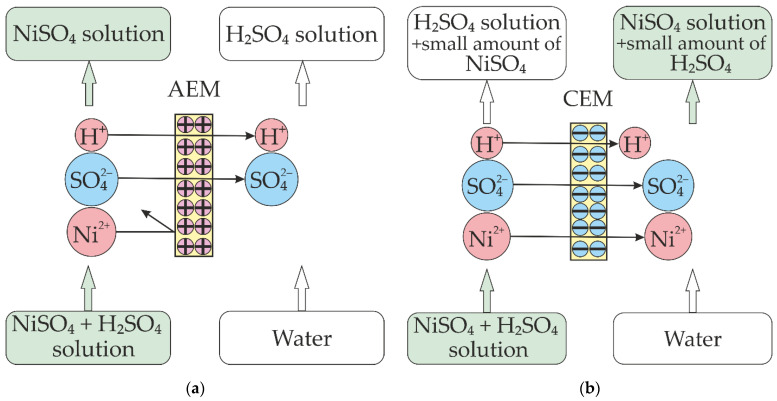
Scheme of ion fluxes though AEM (**a**) and CEM (**b**).

The permeability coefficient for the TWDDA3 membrane was more than 10^-6^ m/s, and the separation factor was more than 25 [46]. The diffusion permeability coefficient for the conventional electrodialysis IEMs depended on the electrolyte solution nature [47] and was significantly lower than for the TWDDA3 membrane. The value of the diffusion permeability coefficient for conventional electrodialysis IEMs was about (1–25)·10^−12^ m^2^/s [35,36,37,41,44]. It is well known that the diffusion flux of acid through AEMs is higher than that of salt [10]. The separation of acid and salt through the anion-exchange membranes was based on this principle (Figure 1a). However, the diffusion flux of acid through the CEMs was lower than that of salt. In this case, one could assume that the receiving solution would be enriched by salt ions while the acid would remain in the feed solution (Figure 1b).

### 2.2. Method of Dialysis

**Figure 2:**
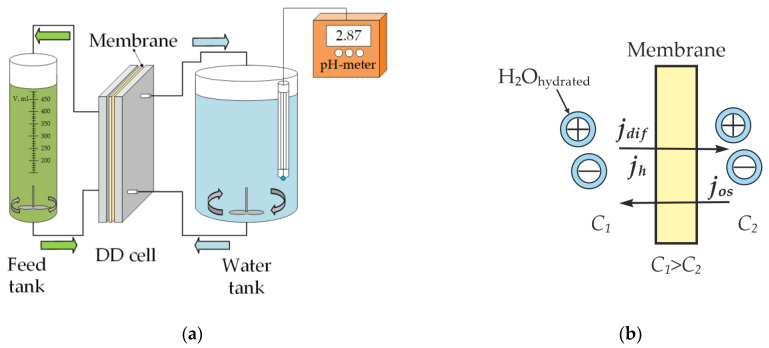
Scheme of DD set-up (**a**) and scheme of electrolyte and water fluxes in the process of DD (**b**).

The study of the DD separation process was carried out in a dual chamber co-current dialyzer (Figure 2a). To ensure flowability, an inert polyethylene mesh-separator was placed in each chamber. The membrane area was 0.01 m^2^. The circulation of the solution was ensured by a Heidolph Pumpdrive 510 peristaltic pump (Heidolph Instruments GmbH & Co, Schwabach, Germany) with a flow rate of 12 L/h through each chamber. The feed solution was a real waste solution from an electroplating facility, containing mostly sulfuric acid and nickel sulfate (Table 2). The processed solution was pumped through the first chamber. The total volume of the processed solution was 0.5 L. Deionized water circulated through the second chamber. To sustain the concentration gradient between solutions separated by an IEM (the driving force of the process), portions of deionized water were changed two times per day at the initial stage and once per day at the final stage. The volume of each portion of deionized water was 5 L. Concentrations of sulfuric acid and nickel sulfate and volumes of the processed solution and the dialysate were measured at certain intervals during the experiment. The sampling interval was about 10–12 h for the electrodialysis membrane (samples 1–5, Table 1) and about 8–10 h for the TWDDA3 membrane. Sulfuric acid concentration was measured by an EasyPlus Automated Titrator (N.V. Mettler-Toledo S.A., Zaventem, Belgium), using acid–base potentiometric titration. Ni^2+^ ion concentration was measured using complexometric titration. The solutions’ densities during the dialysis were measured with an areometer.

The fluxes of nickel sulfate and sulfuric acid through the IEM were determined based on the experimental results according to the formula:

(1) $$j_{i}=\frac{\Delta n^{i}-n_{al}^{i}}{\Delta t\cdot S},$$

where superscript *i* was sulfuric acid or nickel sulfate, Δ*n^i^* was the change in the amount of the *i* substance in the processed solution over a period of time Δ*t*, $n_{al}^{i}$ was the amount of the *i* substance taken with a sample for solution analysis, and *S* was the IEM area. The recovery of sulfuric acid and loss of nickel sulfate ($\chi_{t}^{i}$) were calculated according to the equation

(2) $$\chi_{i}=\frac{n_{0}^{i}-n_{t}^{i}-\sum_{0}^{t}n_{al}^{i}}{n_{0}^{i}-\sum_{0}^{t}n_{al}^{i}}\cdot 100\%,$$

where *t* was the time required for reaching $\chi_{i}$ and $n_{0}^{i}$ and $n_{t}^{i}$ were the amount of the substance of the *i*-component in the processed solution at the initial moment and at *t* time, respectively. The loss of nickel sulfate was the portion of nickel sulfate transferred with sulfuric acid flux through the membrane into the water. The average fluxes of sulfuric acid and nickel sulfate ($\bar{j_{i}}$) in *t* time were calculated as:

(3) $$\bar{j_{i}}=\frac{n_{0}^{i}-n_{t}^{i}-\sum_{0}^{t}n_{al}^{i}}{t\cdot S}.$$

During the dialysis process, a diffusion flux of an electrolyte (*j_dif_*) which was directed to an area with a lower concentration occurred due to a chemical potential gradient (Figure 2b). Due to ion hydration phenomena, the transport of ions results in a drag flux of water with the electrolyte (*j_h_*). Therefore, *j_h_* had the same direction as a diffusion flux. In addition, as two solutions with different concentrations were separated by an IEM, an osmotic water flux (*j_os_*), opposite to diffusion flux, occurred. Thus, the total volume of water flux ($j_{H_{2}O}$) to the processed solution chamber was defined as the difference between the osmotic flux and the drag flux:

(4) $$j_{H_{2}O}=j_{os}-j_{h}.$$

Thereby, a change in the processed solution volume is due to two factors: the drag flux of water in hydration shells decreases the volume, while osmotic flux increases it.

The osmotic water flux might be defined as:

(5) $$j_{os}=P_{H_{2}O}\cdot \left(\pi_{s}-\pi_{H_{2}O}\right),$$

where $P_{H_{2}O}$ was the water permeability of the IEM and $\pi_{s}$ и $\pi_{H_{2}O}$ were the osmotic pressures of the processed and dialysate solutions, respectively. The Van’t Hoff equation or Equation (6) could be used to calculate the osmotic pressure:

(6) $$\pi =\frac{RT}{{\bar{V}}_{H_{2}O}}\cdot \nu c\varphi M_{H_{2}O}$$

where π was the osmotic pressure, *R* was the universal gas constant, *T* was the temperature, ${\bar{V}}_{H_{2}O}$ and $M_{H_{2}O}$ were the partial molar volume and molecular weight of the solvent, respectively, *ν* was the Van’t Hoff coefficient, *c* was the molality of the electrolyte (amount of a solute divided by the mass of the solvent), and *φ* was the molal osmotic coefficient [48].

However, the task of defining osmotic pressure values in concentrated solutions, in which properties greatly deviate from ideal solutions, is quite complex. It includes the problem of determining the activity coefficients and osmotic coefficients of electrolytes. The thermodynamic properties of many electrolyte solutions have been defined experimentally, but this data could not be used for solutions containing more than one electrolyte. Furthermore, theoretical derivation is extremely difficult, except in the case of solutions containing either one electrolyte or two binary electrolytes. Thus, work [49] was dedicated to the model ion composition of sulfuric acid and to the evaluation of the osmotic coefficients of solutions with concentrations up to 6 mol/L. The authors used a method based on the modified Debye–Hückel theory. This method was developed in a series of studies including systems containing one or several solutes [50,51,52,53]. It was shown that results obtained using this model were in good agreement with the experimental data. At the same time, this model used a number of fitting parameters and virial coefficients representing forces of ion–ion interactions for a range of electrolytes. However, the application of this method was not verified for a system considered in this study. In [54], osmotic pressures were defined according to the Van’t Hoff equation and model [49] for a solution containing copper and nickel sulfates given that their ratio was a constant value. It was shown that the experimental data of osmotic pressure values in these solutions agrees well with the results obtained using model [49] and did not agree with the results calculated according to the Van’t Hoff equation. The problem of defining osmotic coefficients and activity coefficients for individual electrolyte solutions, taking hydration and ion association into account, were solved in [55,56]. In some studies, the authors disregarded the presence of the second component in the solution and considered the parameters for a solution with one solute [19].

In general, a precise calculation of all the ion forms in concentrated solutions containing several components was a difficult task requiring separate consideration. Thus, the total concentration of hydrogen ions, which appear as a proton and a hydro-sulfuric anion in solution, and nickel ions were defined in this study. The thermodynamic properties of the system, such as osmotic pressure or degree of dissociation, were not determined.

The estimation of the total water flux was calculated based on the following reasoning. The weight loss of the processed solution was equal to the sum of the masses of water, sulfuric acid, and nickel sulfate which transferred to the dialysate. The total water flux was calculated using the formula:

(7) $$j_{H_{2}O}=\frac{\left(\Delta V\cdot \rho_{s}-m_{H_{2}SO_{4}}-m_{NiSO_{4}}\right)}{\rho_{H_{2}O}\cdot S\cdot \Delta t},$$

where Δ*V* was the change in processed solution volume during the time Δ*t*, *ρ_s_* was the processed solution density, $\rho_{H_{2}O}$ was the water density (1000 kg/m^3^), and $m_{H_{2}SO_{4}}$ and $m_{NiSO_{4}}$ were the masses of sulfuric acid and nickel sulfate which transferred to the dialysate during the time Δ*t,* respectively. The water drag flux was calculated using the formula:

(8) $$j_{h}=\left[j_{H_{2}SO_{4}}\cdot \left(2h_{H^{+}}+h_{SO_{4}^{2-}}\right)+j_{NiSO_{4}}\cdot \left(h_{Ni^{2+}}+h_{SO_{4}^{2-}}\right)\right]\cdot \frac{M_{H_{2}O}}{\rho_{H_{2}O}},$$

where $h_{H^{+}}$, $h_{SO_{4}^{2-}}$ and $h_{Ni^{2+}}$ were the solvation number of the *H^+^*, $SO_{4}^{2-}$, and *Ni*^2+^ ions, respectively. A similar approach to estimate the water drag flux carried in the hydration shell of ions was used in [19]. The adopted solvation numbers were 2 for *H*^+^, 5 for $SO_{4}^{2-}$, and 5 for *Ni*^2+^ [57,58,59,60]. The osmotic flux was calculated as a difference between total water flux and drag water flux using the Formula (4).

Formula (5) and the Van’t Hoff equation could be used to estimate $P_{H_{2}O}$:

(9) $$P_{H_{2}O}=\frac{j_{os}}{R\cdot T\cdot \Delta C},$$

where Δ*C* was the difference in ion concentration between the processed solution and the dialysate, which was calculated using the sulfuric acid and nickel sulfate concentration and the Van’t Hoff coefficients. According to [61], the $P_{H_{2}O}$ was determined by formula:

(10) $$P_{H_{2}O}=\frac{D_{H_{2}O}\cdot C_{H_{2}O,1}^{m}\cdot {\bar{V}}_{H_{2}O}}{R\cdot T\cdot l},$$

where $D_{H_{2}O}$ was the water diffusion coefficient in the membrane and $C_{H_{2}O,1}^{m}$ was the water concentration inside membrane at the feed interface. Formula (10) showed that the water permeability included the membrane thickness. It allowed the membrane water permeability for the reverse osmosis membranes and ultra- and nanofiltration membranes, which had a similar thickness [61], to be compared. However, in the present study IEMs had different thicknesses. It was well known that the diffusion flux could be determined using the following formula:

(11) $$j_{i}=\frac{P_{i}\cdot \Delta C}{l},$$

where *P_i_* was the coefficient of membrane diffusion permeability [61]. Then, the water permeability coefficient ($P_{H_{2}O}^{*}$) was proposed to compare the properties of membranes with different thicknesses. $P_{H_{2}O}^{*}$ could be calculated by the formula

(12) $$P_{H_{2}O}^{*}=\frac{j_{os}\cdot l}{R\cdot T\cdot \Delta C}.$$

## 3. Results

### 3.1. Sulfuric Acid and Nickel Sulfate Transfer through Membranes

**Figure 3:**
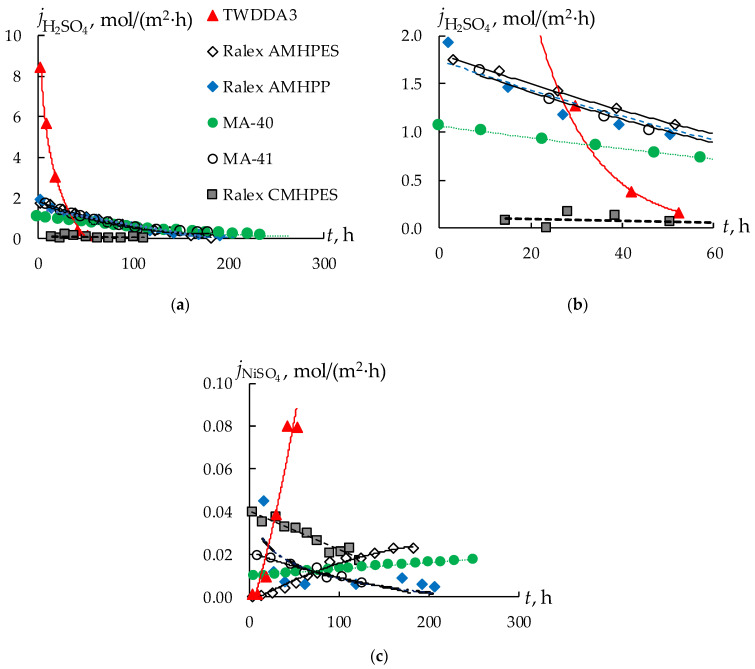
Kinetic dependence of the sulfuric acid fluxes (**a**), the initial section of this dependence (**b**) and nickel sulfate fluxes (**c**) for membranes during DD. The data caption on all figures corresponds to the caption given in (**a**).

The sulfuric acid and nickel sulfate fluxes defined in the process of DD for IEMs of different types are shown in Figure 3. Analysis of the obtained results shows that an essential difference occurs in the values of fluxes passing through AEMs and a CEM. The sulfuric acid practically is not transferred through the CEM in the presence of comparable nickel sulfate flux (Figure 2, grey squares). The obtained results are in good agreement with the results of studying the diffusion permeability of heterogeneous CEMs. In [47], the authors showed that sulfuric acid flux is lower than sodium chloride flux in the concentration range used in the present study. During dialysis, a decrease in the flux of nickel sulfate through the CEM from 0.04 to 0.02 mol/(m^2^ h) was observed, while its concentration in the processed solution decreased by four times, from 0.018 mol/L to 0.07 mol/L. The flux of sulfuric acid also decreased from 0.09 to 0.01 mol/(m^2^·h), which was accompanied by a decrease in the concentration of sulfuric acid in the processed solution to 0.9 mol/L. Thus, these components cannot be separated, which made it impractical to use a CEM for this process.

The highest values of the flux and the transfer rate of sulfuric acid are naturally observed for the thinnest dialysis TWDDA3 membrane (Figure 3a). As the membrane thickness increases, the H_2_SO_4_ transfer rate decreases. The Ralex AMHPES, Ralex AMHPP, and MA-41 membranes, which differ only in the material of the reinforcing mesh and the degree of grinding of the ion-exchange resin, have very similar characteristics in the DD process. An interesting fact is that the rate of transfer of sulfuric acid through the MA-40 membrane is the lowest compared to all other AEMs. This fact could be explained by the higher ion-exchange capacity value for the MA-40 membrane than for all other studied AEMs. Moreover, all the ionogenic groups of the MA-40 membrane are in the protonated state in acidic solutions, in contrast to solutions with a neutral or alkaline environment. This might lead to a significant difficulty in the transport of co-ions through the membrane compared to membranes with a lower exchange capacity, which explains the low diffusion transfer rates.

**Figure 4:**
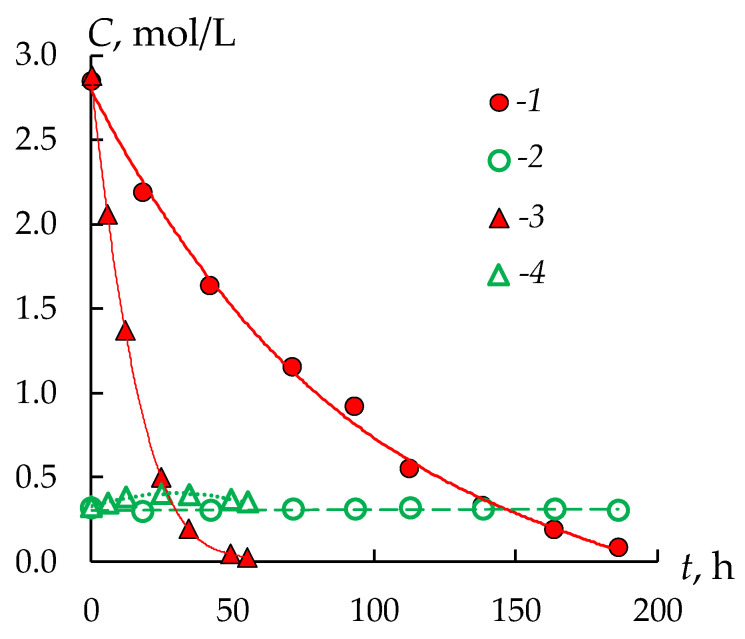
Kinetic dependence of the concentrations of sulfuric acid (1, 3) and nickel sulfate (2, 4) in the processed solution during the DD process with the MA-41 membrane (1, 2) and TWDDA3 one (3, 4).

The nickel sulfate flux through the AEMs does not exceed 0.025 mol/(m^2^ h), except for the dialysis TWDDA3 membrane (Figure 3c). For this membrane, an almost linear increase with time in the flux of nickel sulfate is observed up to 0.08 mol/(m^2^ h) (Figure 3c), which is significantly higher compared to all other samples. The dependences of the nickel sulfate fluxes on time for almost all membranes have an increasing character. A decrease in the concentration of sulfuric acid in the processed solution because of its transfer through the membrane leads to an increase in the nickel sulfate flux. This effect is especially pronounced for the TWDDA3 membrane. The concentration of sulfuric acid in the processed solution decreases very quickly (Figure 4, curve 3). The concentration of H_2_SO_4_ is 6–9 times higher than the concentration of nickel sulfate at the beginning of the experiment. After 27 h their concentration becomes almost equal, and afterwards the concentration of nickel sulfate in the solution exceeds the concentration of sulfuric acid. As a result, the transport of nickel sulfate increases (Figure 4, curves 2, 4).

The form of the kinetic dependence of the concentrations of sulfuric acid and nickel sulfate in the processed solution during the DD process with all AEMs is similar; only the rate of decrease in the concentration of sulfuric acid differs (Table 3). Thus, the time to reach the equal concentrations of sulfuric acid and nickel sulfate in the processed solution for the MA-41 membrane is about 150 h, and for the TWDDA3 membrane it is about 27 h (Figure 4). At the same time, the flux of nickel sulfate remains significantly lower than the flux of sulfuric acid for all AEMs (Figure 3, Table 3).

**Figure 5:**
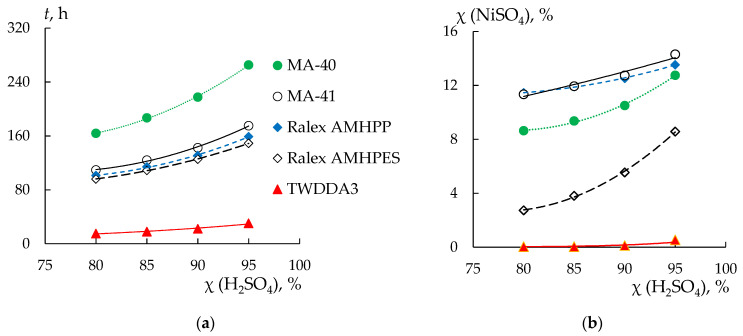
The time of reaching 80–95% recovery of sulfuric acid (**a**) and the loss of nickel sulfate (**b**) for the studied membranes. The signature of the data in the figures corresponds to the signature given in (**a**).

Based on the obtained experimental data, the time of reaching 80, 85, 90, and 95% recovery of sulfuric acid, the loss of nickel sulfate by this time, and the average fluxes of sulfuric acid and nickel sulfate are calculated (Table 3, Figure 5). The time of achieving the desired levels of sulfuric acid recovery is similar for both Ralex membranes and the MA-41 membrane and is about 100–160 h. The longest recovery is achieved on the MA-40 membranes (from 160 to 270 h) and the dialysis TWDDA3 membrane is the most effective. The time of reaching 95% sulfuric acid recovery is 30.5 h.

For all membranes except the Ralex AMHPES membrane, nickel sulfate loss increases insignificantly with an increase in the degree of sulfuric acid recovery from 80 to 95% (Figure 4b). At the same time, the highest loss of nickel sulfate is observed for the MA-41 (up to 14%) and Ralex AMHPES (11–13%) membranes, the lowest for the TWDDA3 sample (does not exceed 1%) (Figure 5b, Table 3). However, for the Ralex AMHPES membrane, a sharp increase in the loss of nickel sulfate by almost three times (from 3 to 9%) is observed with an increase in the degree of sulfuric acid recovery from 80 to 95%.

The values of the average fluxes of sulfuric acid naturally decrease with increasing dialysis time due to a decrease in the concentration gradient between the chambers. The change in the values of the average fluxes of nickel sulfate from time for different membranes does not have a general pattern (Table 3). With the increase in dialysis time, the flux of nickel sulfate is expected to increase due to a decrease in the flux of sulfuric acid and an increase in the concentration of nickel sulfate in the processed solution. Indeed, for the TWDDA3 and Ralex AMHPES membranes, an increase in the average flux of nickel sulfate by 10 and seven times is observed with an increase in the degree of sulfuric acid recovery from 80 to 95%. In this case, the increase in the concentration of nickel sulfate in solution is not proportional to the change in flux. However, there is a tendency towards a decrease in the average flux for the remaining three membranes. The decrease is most pronounced for the Ralex AMHPP and MA-41 membranes. At the same time, the concentration of nickel sulfate increases with the increase in the time of the dialysis process for the TWDDA3 and Ralex AMHPES membranes. Separately, it should be noted that in the case of the MA-41 membrane, the concentration of nickel sulfate in the processed solution increases very slightly. Therefore, if for the rest of the membranes there is an increase in concentration from the initial value of 0.30 M to 0.37–0.40 M, for the MA-41 membrane the concentration of nickel sulfate will not exceed 0.31 M. Such features of the change in the value of the average flux of nickel sulfate and the change in the content of nickel sulfate connected with water transport in the DD process is discussed below. Thus, the task of separating sulfuric acid and nickel sulfate is achieved using all AEMs, but the most effective is the thin dialysis TWDDA3 membrane. However, the use of a cation-exchange membrane in the DD process does not allow the separation of sulfuric acid and nickel sulfate.

### 3.2. Water Transport during Diffusion Dialysis

The phenomenon of “positive” and “negative” osmosis is described in [62]. It consists in an abnormally high transfer of the solvent or in a transfer of a solvent from a more concentrated solution to a more diluted one through an IEM during dialysis. An electric potential difference is formed between the outer boundaries of the membrane due to the difference in the mobilities of the counter- and co-ions. It leads to solution flow inside the membrane. This flow will be directed towards the concentrated solution in the case of a more mobile counter-ion (“positive osmosis”). Conversely, in the case of a more mobile co-ion, the direction of the solution flow inside the membrane will be towards the diluted solution (“negative osmosis”).

**Figure 6:**
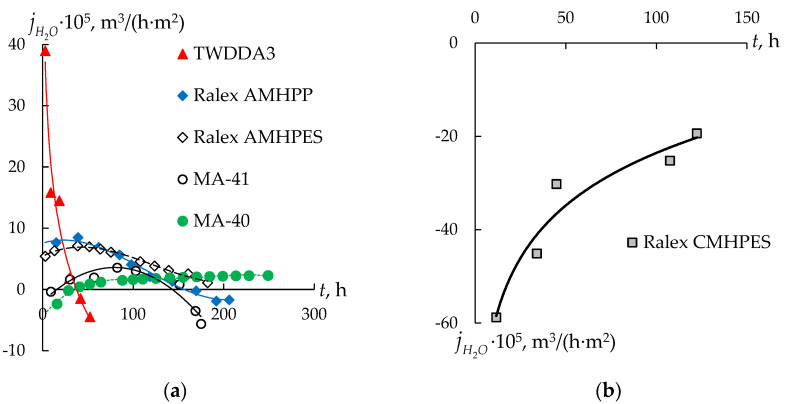
Total water flux through different types of AEMs (**a**) and CEM (**b**) during dialysis.

Figure 6 shows the total water flux during dialysis for the studied membranes. Negative flux values indicate that the osmotic flux exceeds the drag water flux and that the amount of solvent in the processed solution increases. It is observed in the case of a CEM (Figure 6b).

It is shown that during the entire process of dialysis, osmosis exceeds the reverse drag water flux. The value of the osmotic flux decreases by three times, which is associated with a decrease in the concentration gradient between the processed solution and the diluted one. At the same time, the observed result does not contradict the concept of “positive and negative osmosis”. In the case of a CEM, the co-ion is less mobile, and thus the direction of the flow of the solution inside the membrane is directed towards a more concentrated solution. Thus, the observed decrease in the concentration of sulfuric acid in the processed solution is the result of both diffusion transfer through the membrane and dilution due to the osmotic transfer of water from a diluted to a concentrated electrolyte solution.

In the case of AEMs, the dependences have a different shape. For the dialysis TWDDA3 membrane, there is a decrease in the total water flux by more than 21 times up to negative values. Negative values are reached after 40 h of DD, which corresponds to 98% removal of sulfuric acid from the processed solution and a residual concentration of 0.022 mol/L for sulfuric acid and 0.35 mol/L for nickel sulfate. At the same time, the dependence of the nickel sulfate concentration on the dialysis time has an extremum, and the time of reaching the maximum concentration of nickel sulfate in the processed solution (about 25 h) apparently corresponds to the moment when the osmotic flux begins to increase and dominate over the drag water flux and “negative osmosis” due to the higher mobility of *H^+^* co-ions compared to sulfate anions. In general, during dialysis, there is a slight increase in the concentration of nickel sulfate, associated with a decrease in the amount of solvent in the processed solution.

For the MA-40 membrane, there is an increase in the total water flux with the time of the process from negative values at the beginning of the experiment, when the osmotic transfer is higher than the transfer of water with solutes, to positive ones. Until the end of the experiment, the total water flux remains positive. It indicates the predominant contribution of “negative osmosis” and drag water flux. It leads to the transfer of the solvent from a more concentrated to a less concentrated solution against the osmotic flux. For other AEMs (the Ralex AMHPES, Ralex AMHPP, and MA-41 membranes), the dependence of the total water flux on the time of the dialysis process is extremal. At the same time, a significant difference in the values of the total water flux through AEMs with similar composition and structure (the MA-41 membrane and both Ralex ones) is observed. Thus, in the case of the MA-41 membrane, the maximum value of the total water flux is 3.5 ×·10^−5^ m^3^/(m^2^·h), while for the Ralex AMHPES and Ralex AMHPP membranes it is 7.1 ×·10^−5^ and 8.5·10^−5^ m^3^/(m^2^·h), respectively. It should also be noted that the final composition of the processed solution is similar for all the studied membranes.

The kinetic factor may be of great importance: due to the high rate of sulfuric acid transfer through the thin dialysis TWDDA3 membrane, the counter-osmotic flux of water is inhibited. Another extreme case is the Ralex CMHPES membrane. The total fluxes of sulfuric acid and nickel sulfate through the Ralex CMHPES membrane are very low. The osmotic flux leads to a significant increase in the amount of the solvent in the processed solution. The remaining membranes occupy an intermediate position, and the amount of the total water flux correlates with the amount of sulfuric acid flux through them.

**Figure 7:**
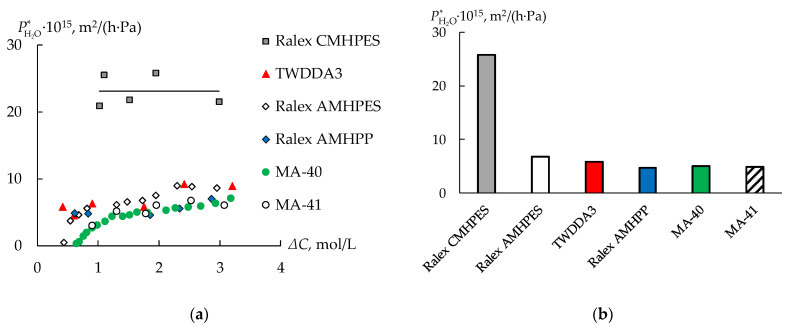
Water permeability coefficient of the membranes depending on the concentration difference (**a**) and values of the membranes water permeability coefficient at Δ*C* = 1.75 mol/L (**b**).

Estimation of the water permeability coefficient of the studied membranes shows that, as expected, the highest values are found for the CEM (Figure 7). In this case, the dependence of the water permeability coefficient on the concentration difference for the CEM is practically absent. Thus, the dependences for AEMs are increasing.

Comparison of the water permeability of AEMs shows that the dialysis TWDDA3 membrane and Ralex AMHPES membrane have a slightly higher permeability than others. However, the values of the water permeability of all AEMs are similar. The total water flux through the cation- and anion-exchange membranes has the opposite direction. It leads to an increase in the volume of the processed solution in the case of the Ralex CMHPES membrane and a decrease in the concentration of nickel sulfate and sulfuric acid. The total water flux through an anion-exchange membrane is directed to the water chamber, which contributes to an increase in the electrolyte concentration in the processed solution and improves the efficiency of DD.

## 4. Conclusions

DD of the real waste solution of an electroplating facility, containing mostly sulfuric acid and nickel sulfate, using different ion-exchange membrane types is studied. The sulfuric acid and the nickel sulfate fluxes are similar for the cation-exchange membrane. Furthermore, the total water flux through the cation-exchange membrane is directed opposite to the diffusion fluxes, and the volume of the processed solution is increased by three times. Thus, the use of a cation-exchange membrane in the DD process does not allow the separation of sulfuric acid and nickel sulfate. However, using the anion-exchange membranes in the DD process provides for the separation of sulfuric acid and nickel sulfate. As expected, the thinnest dialysis membrane is the most effective. For a membrane area of 0.01 m^2^, the time of reaching 95% recovery of sulfuric acid is 30.5 h, which is about five times less than for the next most efficient membrane (Ralex AMHPES). The weak basic anion-exchange membrane MA-40 is less effective than other anion-exchange membranes. The total water fluxes and diffusion fluxes through all the anion-exchange membranes are directed equally for most of the DD time. The values of the water permeability coefficient of ion-exchange membranes are estimated. The highest values of the water permeability coefficient are observed for the cation-exchange membrane. The values of the water permeability coefficient of all anion-exchange membranes are similar.

In general, it is found that the use of all strongly basic anion-exchange membranes makes the separation of sulfuric acid and nickel sulfate by diffusion dialysis possible. A nickel sulfate solution with a sulfuric acid concentration less than 0.1–0.2 mol/L is obtained. Despite the longer time required to achieve a high degree of separation compared to using a dialysis membrane, the use of conventional electrodialysis membranes may be economically justified due to their lower cost.

## Figures and Tables

**Table 1:** The physical–chemical properties of IEMs.

Sample No.	Membrane	Ionogenic Groups	Reinforcing Mesh	*Q,* mmol/g_wet_	*W*, %	*k*_iso_, S/m	Counter-Ion Transport Number	*l*, μm
1	Ralex AMHPES	quaternary ammonium bases	polyester	1.05	66.3	0.42–0.46 [34,35]	>0.95 [31]	550
2	Ralex AMHPP	quaternary ammonium bases	polypropylene	0.97	71.3	-	>0.95 [31]	550
3	Ralex CMHPES	sulfonic groups	polyester	1.16	79.9	0.35–0.40 [34,35]	>0.95 [31]	530
4	MA-40	secondary and tertiary amines	nylon	2.15	53.0	0.08 [33,36]	0.94 [36]	520
5	MA-41	quaternary ammonium bases	nylon	0.54	49.1	0.19–0.28 [35,37]	0.96 [36]	420
6	TWDDA3	quaternary ammonium bases	polyphenylene oxide	0.49	98.3	-	-	145

**Table 2:** The composition of the waste solution *.

Solution Component	Concentration, g/L
H_2_SO_4_	252.3
Ni^2+^	20.9
Zn^2+^	2.3
Fe^2+^	1.08
Cu^2+^	0.90
Total Sn	0.080
Total Sb	0.050
Total Cd	0.045
Total Pb	0.015
Total As	0.003

* Data provided by the plant. The content of metals takes into account all ionic forms in terms of a simple substance.

**Table 3:** Average fluxes of sulfuric acid (mol·m^−2^·h^−1^) and nickel sulfate through various AEMs upon reaching 80–95% recovery of sulfuric acid ($\chi_{H_{2}{\mathrm{SO}}_{4}}$).

$\chi_{H_{2}{\mathrm{SO}}_{4}},\;\%$	80	85	90	95
	**TWDDA3**			
H_2_SO_4_	4.63	4.64	4.48	3.95
NiSO_4_	0.001	0.003	0.004	0.010
	**MA-41**			
H_2_SO_4_	1.02	0.96	0.88	0.77
NiSO_4_	0.016	0.016	0.015	0.010
	**MA-40**			
H_2_SO_4_	0.66	0.62	0.56	0.49
NiSO_4_	0.0003	0.001	0.002	0.003
	**Ralex AMHPES**			
H_2_SO_4_	1.12	1.06	0.98	0.87
NiSO_4_	0.001	0.003	0.004	0.007
	**Ralex AMHPP**			
H_2_SO_4_	1.08	1.03	0.94	0.82
NiSO_4_	0.012	0.011	0.010	0.010

## Data Availability

The data presented in this study are available on request from the corresponding author.

## List of Symbols and Abbreviations

Subscripts and superscripts:

Subscript “0” denotes that the magnitude refers to the initial time of the experiment;

Subscript “*t*” denotes that the magnitude refers to the time *t* of the experiment;

Subscript “*al*” denotes that the magnitude refers to the sample for solution analysis;

Subscript or superscripts “*i*” denotes that the magnitude refers to the *i*-component;

Superscripts “s” denotes that the magnitude refers to the processed solution.

Abbreviations:		
AEM	anion-exchange membrane	
CEM	cation-exchange membrane	
DD	diffusion dialysis	
ED	electrodialysis	
IEM	ion-exchange membrane	
Greek Letters		
Parameter	Description	Dimension
Δ	change in a variable	
Χ	recovery of sulfuric acid or loss of nickel sulfate	%
Π	osmotic pressure	Pa
*Φ*	molal osmotic coefficient	
*Ν*	Van’t Hoff coefficient	
ρ	density	$\mathrm{kg}/m^{3}$
English letters		
Parameter	Description	Dimension
*C*	molar concentration	$\mathrm{mol}/m^{3}$
C	molality of electrolyte	$\mathrm{mol}/{\mathrm{kg}}_{H_{2}O}$
$h_{H^{+}}$, $h_{SO_{4}^{2-}}$ and $h_{Ni^{2+}}$	the solvation number of the *H^+^*, $SO_{4}^{2-}$ and *Ni^2+^* ions, respectively	${\mathrm{mol}}_{H_{2}O}/{\mathrm{mol}}_{i}$
$j_{H_{2}O}$	total water flux	$m^{3}/\left(m^{2}\cdot h\right)$
$j_{os}$	osmotic water flux	$m^{3}/\left(m^{2}\cdot h\right)$
$j_{h}$	drag flux	$m^{3}/\left(m^{2}\cdot h\right)$
*j_i_*	fluxes of *i* electrolyte	$\mathrm{mol}/\left(m^{2}\cdot h\right)$
$\bar{j_{i}}$	average flux of *i* electrolyte in time *t*	$\mathrm{mol}/\left(m^{2}\cdot h\right)$
***k*_iso_**	the membrane gel phase conductivity	S/m
*l*	membrane thickness	m
*M*	molecular weight	kg/mol
*m*	mass	kg
*n*	amount of a substance	mol
$P_{H_{2}O}$	water permeability of the IEM	$m^{3}/\left(m^{2}\cdot h\cdot \mathrm{Pa}\right)$
$P_{H_{2}O}^{*}$	water permeability coefficient	$m^{2}/\left(h\cdot \mathrm{Pa}\right)$
*Q*	ion-exchange capacity	mmol/g
*R*	universal gas constant	$J/\left(\mathrm{mol}\cdot K\right)$
*S*	membrane area	m^2^
*T*	temperature	K
*t*	time	h
*V*	volume	m^3^
$\bar{V}$	partial molar volume	$m^{3}/\mathrm{mol}$

## Appendix A

The membrane pretreatment procedure was carried out as follows: surface treatment with carbon tetrachloride for degreasing; conditioning in ethanol within 6 h to remove residues of monomers and oligomers from the ion-exchange resin; conditioning for 48 h in the solutions of saturated NaCl, 100 g/L NaCl and 30 g/L NaCl successively; washing the membranes with deionized water and treating them with 10% HCl for 48 h to transfer the ionogenic groups to the H^+^- form in the case of cation-exchange membrane and 10% NaOH to transfer the ionogenic groups to the OH^−^- form in the case of anion-exchange ones; washing the membranes with deionized water until the neutral reaction of methylene orange or phenolphthalein for cation- and anion-exchange membranes respectively; treating anion-exchange membranes with 10% H_2_SO_4_ for 48 h to transfer the ionogenic groups to the sulfate form and washing with deionized water; treating the membranes with the processed solution for 24 h before carrying out the experiment.

TWDDA3 membrane had not been treated with CCl_4_, ethanol and alkaline solution according to the recommendations of the manufacturer.

Ion-exchange capacity (*Q*, mmol/g_wet_) determination was carried out by the following method: the membranes were weighed (*m_wet_*, g); the cation-exchange membranes in the H^+^- form were immersed with a known volume of 0.1 M NaOH (*V*, mL) and the anion-exchange in the OH^−^- form were immersed with a known volume of 0.1 M HCl (*V*, mL); after 24 h, acid-base titrations of contact solutions of NaOH and HCl (*V_al_*, mL) with methylene orange or phenolphthalein was performed; ion-exchange capacity was calculated as the difference of initial (*C*, mol/L) and final concentration of the contact solution divided by the mass of the wet membrane by the formula

$$Q=\frac{\left(C-\frac{C_{titr}\cdot V_{titr}}{V_{al}}\right)}{m_{wet}}\cdot V,$$

where *C* was the initial concentration of NaOH or HCl solution, *C_titr_* and *V*_titr_ were the concentration and equivalence volume of titrant.

Water content (*W*, %) was carried out as follows: the membranes was washed by deionized water before the procedure until the water conductivity was constant after the 24 h bathing of the membrane, then the membrane was weighed (*m_wet_*, g) and dried at a temperature of 105 °C during 1 h, then the membrane was weighed (*m*_1*dry*_) and re-dried the membranes at a temperature of 105 °C during 1 h, then the membrane was weighed (*m*_2*dry*_), the procedure was repeated until the masses of the dry membrane matched (*m*_1*dry*_ *= m*_2*dry*_ *= m_dry_*), then the water content calculated by the formula

$$W=\frac{m_{wet}-m_{dry}}{m_{dry}}\cdot 100\%$$

The measurement error in determining the water content was no more than 5%.
